# A Telemedicine-Guided Self-Collection Approach for PCR-Based SARS-CoV-2 Testing: Comparative Study

**DOI:** 10.2196/32564

**Published:** 2022-01-04

**Authors:** Silvia Würstle, Johanna Erber, Michael Hanselmann, Dieter Hoffmann, Stanislas Werfel, Svenja Hering, Simon Weidlich, Jochen Schneider, Ralf Franke, Michael Maier, Andreas G Henkel, Roland M Schmid, Ulrike Protzer, Michael Laxy, Christoph D Spinner

**Affiliations:** 1 Department of Internal Medicine II, School of Medicine University Hospital rechts der Isar Technical University of Munich Munich Germany; 2 Department for Sport and Health Sciences Professorship of Public Health and Prevention Technical University of Munich Munich Germany; 3 School of Medicine / Helmholtz Zentrum München Institute of Virology Technical University of Munich Munich Germany; 4 Department of Nephrology, School of Medicine University Hospital rechts der Isar Technical University of Munich Munich Germany; 5 Siemens AG Munich Germany; 6 Department of Information Technology School of Medicine, University Hospital rechts der Isar Technical University of Munich Munich Germany; 7 German Centre for Infection Research (DZIF) Partner site Munich Germany

**Keywords:** self-sampling, telemedicine, test strategy effectiveness, simulation model, SARS-CoV-2, COVID-19

## Abstract

**Background:**

Large-scale, polymerase chain reaction (PCR)-based SARS-CoV-2 testing is expensive, resource intensive, and time consuming. A self-collection approach is a probable alternative; however, its feasibility, cost, and ability to prevent infections need to be evaluated.

**Objective:**

This study aims to compare an innovative self-collection approach with a regular SARS-CoV-2 testing strategy in a large European industrial manufacturing site.

**Methods:**

The feasibility of a telemedicine-guided PCR-based self-collection approach was assessed for 150 employees (intervention group) and compared with a regular SARS-CoV-2 testing approach used for 143 employees (control group). Acceptance, ergonomics, and efficacy were evaluated using a software application. A simulation model was implemented to evaluate the effectiveness. An interactive R shiny app was created to enable customized simulations.

**Results:**

The test results were successfully communicated to and interpreted without uncertainty by 76% (114/150) and 76.9% (110/143) of the participants in the intervention and control groups, respectively (*P=.*96). The ratings for acceptability, ergonomics, and efficacy among intervention group participants were noninferior when compared to those among control group participants (acceptability: 71.6% vs 37.6%; ergonomics: 88.1% vs 74.5%; efficacy: 86.4% vs 77.5%). The self-collection approach was found to be less time consuming (23 min vs 38 min; *P*<.001). The simulation model indicated that both testing approaches reduce the risk of infection, and the self-collection approach tends to be slightly less effective owing to its lower sensitivity.

**Conclusions:**

The self-collection approach for SARS-CoV-2 diagnosis was found to be technically feasible and well rated in terms of acceptance, ergonomics, and efficacy. The simulation model facilitates the evaluation of test effectiveness; nonetheless, considering context specificity, appropriate adaptation by companies is required.

## Introduction

Numerous campaigns for COVID-19 vaccination have been initiated worldwide, but the pandemic continues to spread. Emerging variants of SARS-CoV-2, as well as reports of breakthrough infections, underline that public health mitigation measures, including testing strategies, need to be continued. In terms of sensitivity, nucleic acid amplification tests (NAATs) are standard for SARS-CoV-2 detection in respiratory samples obtained by medical personnel (ie, regular testing approach) [1]. However, the implementation of professional, large-scale routine testing is limited owing to high organizational costs and intensive efforts entailed. The authorization of lateral-flow SARS-CoV-2 antigen tests enables the implementation of self-testing strategies (ie, self-testing approach), which improves the turnaround times of test results. Due to the absence of amplification steps, the analytic sensitivity of lateral-flow antigen tests is substantially inferior to that of a NAAT-based approach, particularly when the viral load is low in early or late stages of disease progression [2].

A self-collection approach based on NAAT performed on self-collected swabs could combine the advantages of regular testing and self-testing approaches. Previous studies have shown that the sensitivity of SARS-CoV-2 polymerase chain reaction (PCR) testing using self-collected swabs was comparable to swabs collected by health care professionals, with an acceptable impact on the test sensitivity [3-7]. Self-collection reduces the use of resource-intensive testing centers and personal protective equipment, and it eliminates the requirement of swab collection by medical personnel. Furthermore, the willingness of individuals to undergo testing might be increased as the time required for testing is expected to be lesser, and self-collected swabs are considered to be more convenient for the operator [8]. Structured evaluations of different testing strategies are required to compare their feasibility, costs, and infection-prevention capabilities.

In this prospective, two-arm feasibility study, we aimed to compare a telemedicine-guided self-collection approach with a regular testing approach involving a sample collected by a health care professional for PCR-based SARS-CoV-2 diagnostics, primarily focusing on the feasibility, and secondarily on the acceptance, ergonomics, and efficacy of the testing strategy implemented onsite at a large European industrial manufacturing company in Germany. For the self-collection approach, we developed a telemedicine-guided approach, which included obtaining electronic consent, electronic registration, and communication of the SARS-CoV-2 PCR test result. Testing approaches in companies aim to reduce the infection risk arising from undetected but infectious employees. In this study, none of the SARS-CoV-2 PCR tests performed in the employees returned positive. We performed health economic modeling to analyze potential effects of different testing strategies and developed a shiny app to enable people to run simulations using different medical assumptions.

## Methods

### Study Design and Participants

**Figure 1:**
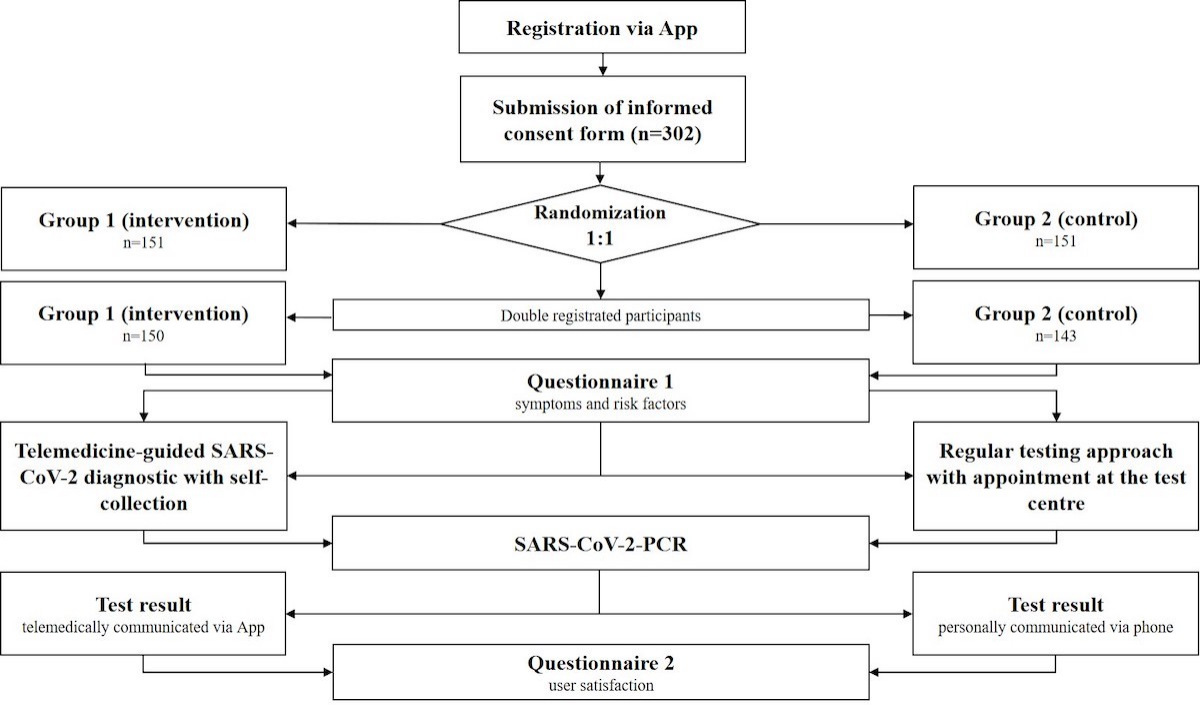
Process flow. Upon registration and informed consent, participants were randomized into a self-collection (intervention) and regular testing (control) group. Swabs from participants of both groups were submitted to the study center and SARS-CoV-2 polymerase chain reaction (PCR) was performed. Questionnaire on the symptoms and risk factors was completed before conducting the procedure, whereas the questionnaire on user satisfaction was completed after the study procedure. The term ‘app’ refers to the software application principa (PlanOrg GmbH, Jena, Germany).

The process flow of this prospective, interventional, open-label, controlled, two-arm feasibility trial is illustrated below (Figure 1). Between November 11, 2020, and December 11, 2020, all employees of Siemens F80, SYKATEC GmbH, and Valeo Siemens eAutomotive Germany GmbH were invited to participate in this study (see Figure S1 in Multimedia appendix 1 for the advertisement flyer). The main inclusion criteria were the ability to download and use the user application of the software app principa (PlanOrg GmbH), which is part of the hospital information system and clinical workplace system at the University Hospital rechts der Isar (Technical University of Munich, Germany). The app could be downloaded using a study-specific quick response (QR) code with a deep link to the Android or Apple store. Upon registration, electronic consent was obtained from all participants, following which they were randomized 1:1 into two study groups using the Java function SecureRandom (algorithm: SHA1PRNG). Participants in group 1 (intervention group; telemedicine-guided SARS-CoV-2 diagnostic testing with self-collection) were asked to collect a prepacked self-collection kit, including the United Nations’ recommendation of dangerous goods (UN 3373) compliant packaging kit (cardboard and container) with a prepaid shipping label, printed instructions (see Figure S2 in Multimedia Appendix 1), and a swab (FLOQSwabs 552C Regular Flocked Swab with an 80-mm breakpoint, Copan). The risk factors for and the symptoms of SARS-CoV-2 infection were self-reported using the app (see Table S1 in Multimedia Appendix 1). A short explanatory video was made available in the app for demonstration to the participants (see Multimedia Appendix 2). Participants self-collected an oropharyngeal swab, scanned the individualized unique code of the shipping kit, and mailed the kit to the study center at University Hospital rechts der Isar for analysis. SARS-CoV-2 PCR test results were provided via the app; in addition, the PDF report could be exported for personal use. Group 2 participants (control group; regular testing approach via appointment at the test center) were asked to book an appointment via phone for SARS-CoV-2 testing at a test center located at the study site. After assessment of SARS-CoV-2–specific risks and symptoms by a staff member, a nasopharyngeal sample was collected (REST Clinical Virus Transport Medium [CTM] swab, Rapid & Easy System Technology; Noble Biosciences, Inc.) by trained medical staff. The samples were shipped to the study center and processed as described for group 1. SARS-CoV-2 PCR test results were exclusively provided by phone and without any hard copy. Upon communication of the test results, participants in both groups were asked to evaluate the respective testing strategy and app by using a visual slider on a 7-point Likert scale (0=strong disagreement, 6=strong agreement) within the app. The questionnaire items were based on the National Aeronautics and Space Administration (NASA) Task Load Index [9] for evaluating the effort and the Website Analysis and Measurement Inventory for evaluating user satisfaction. The time required for the entire sample collection procedure was assessed in minutes. The completion of the questionnaire was facultative.

### Ethics Approval

This study was approved by the ethics committee at the Technical University of Munich, School of Medicine, University Hospital rechts der Isar, Munich, Germany (approval 603/20-SH) and conducted in accordance with the Declaration of Helsinki.

### Primary and Secondary End Points

The primary end point was the proportion of participants for whom self-collection, virological diagnosis, and reporting of the test result were successfully conducted, and the statements made by the participants in response to the questionnaire did not indicate any uncertainty with respect to interpretation of the test results (see Table S2 in Multimedia Appendix 1 for details). The secondary end points were (1) the proportion of participants for whom virological findings were available but uncertainty regarding the interpretation was reported, and (2) patient-reported outcomes, including acceptance, ergonomics, and efficacy. The statements of group-specific questionnaires were assigned to these three outcomes (see Table S4 in Multimedia Appendix 1). Threshold values were defined to transfer the ratings into a dichotomous scale (favorable rating >3 points; unfavorable rating ≤3 points). A favorable rating for more than 70% of all the statements was interpreted as a satisfactory outcome.

### Diagnostic Procedures

All virological diagnostics were performed by the expert staff of the Institute of Virology, Technical University of Munich. Nucleic acids were extracted using the mSample Preparation System DNA kit (Promega), and a standard protocol was followed on an m2000sp device for RNA and DNA extraction (Abbott). SARS-CoV-2_N1 and SARS-CoV-2_N3 primer and probe sets were used for amplification on an ABI 7500 real-time PCR cycler (Thermo Fisher Scientific), following the protocol of the Division of Viral Diseases, National Center of Immunization and Respiratory Diseases, Centers for Disease Control and Prevention (accreditation authority No. D-ML-14063-02-00) [10].

### Sample Size Calculation

For calculation of the sample size, we assumed that 50% of the participants met the criteria for the primary end point. A confidence level of 95% and a specified CI of 0.08 resulted in 151 participants per group (n=302). Dropouts were not considered because they were represented in the fraction of the participants to whom the test results were not successfully communicated. Recruitment was stopped when the calculated sample size was reached.

### Simulation Model for Evaluation of the Effectiveness of Test Strategy

We developed a simulation model that facilitates evaluation of the effectiveness of distinct COVID-19 test strategies. We modeled the risk of infection that arises from individuals with SARS-CoV-2 infection who do not present typical COVID-19 symptoms. This implies that the model solely focuses on the risk of infection that arises from undetected, presymptomatic, and asymptomatic COVID-19 cases. Test strategies that are targeted on individuals without COVID-19 symptoms represent a measure taken to reduce this risk. The developed simulation model considers a period of 4 weeks (28 days) and relies on several medical assumptions. Based on expert ratings and a review of the relevant literature, we assumed that COVID-19 symptoms appear on the third day of infection [11,12]. We assumed 20% asymptomatic infections [13] and postulated that infected individuals are infectious for 10 days [14]. Furthermore, we assumed a 7-day incidence rate of 100 and 20% immune individuals. The main outcome of the simulation model was the average number of infectious but undetected individuals working onsite per day, which can be interpreted as a measure of the risk of formation of infection clusters within the company. Considering a scenario without any testing strategy as the benchmark, the model allows us to calculate and compare the measurements of relative risk reduction for different testing strategies. In our simulation model, test strategies are defined by five parameters. For this study, we considered a population of 10,000 individuals (workers) and assumed that 80% of susceptible individuals actually participated in the testing program. In addition, we assumed that participants were tested once per week. We assumed these parameters to be equal for both testing programs used in this study. The other two parameters defining a testing program are closely related to the test performed and are therefore of particular importance in this study. The first parameter is test sensitivity, which is supposed to be higher in the case of the regular testing approach. Prior research indicates a sensitivity of 90% for the regular testing approach and 80% for the self-collection approach [5-7,15]. However, because the exact difference remains unknown, we report simulation results for four different levels of test sensitivity: 70%, 80%, 90%, and 100%. The second parameter that is relevant in this study is the number of days taken to communicate the test results to the program participants (ie, turnaround time). Unfortunately, the trial conducted does not provide a clear indication of the turnaround time of both testing approaches. In fact, the turnaround times may differ between the two approaches. Therefore, we ran our simulations for four different turnaround times: 0, 1, 2, and 3. Because we differentiate between the four sensitivity levels and the four turnaround times, we report the results for a total of 16 simulation scenarios. The comparison of the obtained results allows us to gain insights into the potential effectiveness of the testing approaches. Due to the stochastic nature of our simulation model, the result of one simulation run is subject to uncertainty. To address this uncertainty, we ran each of our 16 simulations 1000 times, and accordingly, the mean of the obtained simulation results is presented.

Few of our assumptions and model input parameters might vary from time to time and/or are context specific. Therefore, we developed an interactive R Shiny web application (R Studio version 4.0.5; R Foundation for Statistical Computing), which enables users to customize the model input parameters and run simulations on their own. The developed simulation tool is integrated in a website. The website also contains a detailed description of the simulation model and displays the R function written and used to run the simulations in this study.

It must be noted that the potential reduction in the risk implied by the simulation results is only applicable if the employees follow hygiene standards as if there were no testing programs implemented at their company. Furthermore, large-scale testing of asymptomatic individuals in a situation of low COVID-19 prevalence will lead to a large share of false-positive test results, which might necessitate the unjustified quarantining of many individuals. This potentially leads to a productivity loss and might disturb the employees unnecessarily. The declining rigorousness of the hygiene measures or the lack of trust in the test results could decrease the effectiveness of the testing strategies. Because the effectiveness of risk reduction measures depends on individual compliance, every large-scale testing program should be accompanied by an information campaign explaining the interpretation and the consequences of positive and negative test results. The developed simulation tool was integrated in a website [16].

### Statistical Methods

The distributions of quantitative and qualitative data are presented as the absolute and relative frequencies or medians (range), respectively. Fisher's two-sided exact test or Pearson's chi-squared test were performed on the categorical variables, and Wilcoxon rank-sum test was performed on quantitative parameters. Statistical hypothesis testing was performed on the two-sided exploratory 0.05 significance levels. RStudio (version 4.0.2; R Foundation for Statistical Computing) was used for all statistical analyses.

### Availability of Data and Material

All self-collection instructions, questionnaires, and comments of the questionnaires are provided in Multimedia Appendices 1 and 2. All raw data are available from the corresponding author on request.

## Results

### Baseline Characteristics of Study Cohort

In this study, 302 employees registered and consented for participation. In all, data of 293 unique participants were available for analysis because 9 employees registered twice, as duplicate registrations were not technically prevented by the app. Groups 1 and 2 consisted of 150 and 143 participants, respectively, of which 21.3% (n=32) and 18.2% (n=26), respectively, were female. The median age for both the groups was 42 years (range: 20-61 years in group 1 and 23-64 years in group 2). The completion rate of the symptom and risk factor questionnaire (see Table S1 in Multimedia Appendix 1) was 64% (n=96) in group 1 and 45.4% (n=65) in group 2. In group 1, 51% (n=49) of the participants reported at least one and 21% (n=20) of the participants reported at least two typical symptoms of COVID-19 (ie, fatigue, tiredness, cough, shortness of breath, rhinitis, loss of smell, loss of taste, sore throat, headache, limb pain, shivering, diarrhea, elevated temperature, and temperature ≥38°C), in contrast to at least one reported symptom in 29% (n=19) and at least two reported symptoms in 2% (n=1) of group 2 participants. This resulted in significantly fewer symptomatic participants in group 2 (*P*=.01) during the assessment. Similarly, group 1 participants reported significantly more symptoms for the last 48 hours and 14 days (*P*<.001). Predefined risk factors (ie, active or past history of smoking, cardiovascular disease, diabetes mellitus, immunosuppressive therapy, and immunodeficiency) for severe COVID-19 were reported by 44% (n=42) of group 1 participants and 60% (n=39) of group 2 participants (*P=.*06).

### Primary Study End Point

SARS-CoV-2 test results were successfully communicated to a total of 270 participants without any significant difference in the results between the two groups (139/150, 92.7% in group 1 vs 131/143, 91.6% in group 2). The results were not successfully communicated to 7.3% (11/150) and 8.4% (12/143) of the participants in groups 1 and 2, respectively (*P=.*91). None of the SARS-CoV-2 PCR results returned positive. The proportion of participants with positive primary study end point (ie, test results were successfully transmitted and participants’ responses to the questionnaire did not indicate uncertainty with respect to the interpretation of the test result; see Table S2 in Multimedia Appendix 1) was 76% (114/150) in group 1 and 76.9% (110/143) in group 2 (*P=.*96). The median age of the participants with positive primary end point was 41 (range 20-61) years in group 1 and 43 (range 23-63) years in group 2.

### Secondary Study End Points

A questionnaire evaluating user satisfaction (see Table S2 in Multimedia Appendix 1) was completed by 73.3% (110/150) of the participants in group 1 (response rate: 87/118, 73.7% for male; 23/32, 71.9% for female participants) and 71.3% (102/143) of the participants in group 2 (response rate: 85/117, 72.6% for male and 17/26, 65.4% for female participants). Of all the participants evaluated for user satisfaction, 16.7% (n=25) in group 1 and 14.7% (n=21) in group 2 indicated uncertainty regarding the test result. The age was comparable to that of participants with a positive primary end point: 42 (range 26-60) years for group 1 (*P=.*30) and 38 (range 24-64) years group 2 (*P=.*24). Furthermore, gender was not significantly related to the reporting of uncertainty about the virological test result obtained in either group (group 1: *P=.*20; group 2: *P*=.99).

Based on the assessment of the responses to the questionnaire, acceptance was favorably rated by 71.6% (78/109), ergonomics by 88.1% (96/109), and efficacy by 86.4% (95/110) of the participants in group 1 (see Table S4 in Multimedia Appendix 1). Age was not significantly associated with the favorable evaluation of any of the outcomes (acceptance: *P=.*41, ergonomics: *P=.*30, efficacy: *P=.*71). Further, the evaluation of outcomes was not significant with respect to gender (women compared with men, acceptance: *P*=1.0, ergonomics: *P=.*73, efficacy: *P=.*73). In group 2, acceptance of the regular testing approach was favorably evaluated by 37.6% (38/101), ergonomics by 74.5% (76/102), and efficacy by 77.5% (79/102) of the participants. The study procedure was estimated to consume an average of 23 (median 15, range 5-90) minutes by participants in group 1 compared with 38 (median 30, range 3-180) minutes for those in group 2 (*P*<.001).

### Analysis of Program Effectiveness

A simulation model was developed to evaluate the effect of COVID-19 test strategies on the infection risk arising from undetected but infectious employees. We ran 16 simulations for four different levels of the test sensitivity and four different turnaround times. The mean relative risk reduction scores and the corresponding 95% CIs for these 16 simulations are tabulated below (Table 1). The results shown in the table can be interpreted as follows: given the assumptions described in the Methods section, a test strategy using a SARS-COV-2 PCR test with a sensitivity of 90% and featuring a turnaround time of 1 day has the potential to decrease the risk of infection posed by undetected but infectious workers onsite by 17.89%. All other aspects remaining constant, a higher test sensitivity or a lower turnaround time increases the effectiveness of the testing strategy. Assuming that the turnaround time is comparable for both the regular approach and the self-collection approach, one might conclude that the regular approach is slightly more effective than the self-collection approach. Depending on the turnaround time selected, the difference in the relative risk reduction score ranges between 1.21% and 3.29%. The self-collection approach would be more effective than the regular testing approach, if the turnaround time in the self-collection approach is 1 day lesser than that of the regular approach. To enable people to run simulations with different medical assumptions and to evaluate alternative testing strategies, we developed an interactive R Shiny app [16].

**Table 1:** Simulation results for relative risk reduction and impact of test sensitivity and turnaround time. Each cell of the table depicts the mean of 1000 relative risk reduction scores and the corresponding CI. In each simulation run, a population of 10,000 individuals was considered. The assumptions (see Methods section) include that 80% of the susceptible individuals participate in the testing program once a week. Turnaround time refers to the time taken to communicate the test result after testing.

Turnaround time (days)	Relative risk reduction across various levels of test sensitivity (%), mean (95% CI)			
	100%	90%	80%	70%
0	27.64 (27.24-28.05)	25.22 (24.81-25.63)	21.93 (21.53-22.32)	19.28 (18.91-19.65)
1	20.08 (19.72-20.43)	17.89 (17.53-18.24)	15.75 (15.40-16.08)	14.02 (13.68-14.35)
2	14.13 (13.81-14.44)	12.72 (12.41-13.02)	11.61 (11.32-11.91)	9.83 (9.54-10.11)
3	10.51 (10.25-10.77)	9.68 (9.43-9.93)	8.47 (8.23-8.71)	7.27 (7.06-7.49)

## Discussion

This study aimed to compare the feasibility, acceptance, ergonomics, and efficacy of an innovative telemedicine-guided self-collection approach with a regular SARS-CoV-2 testing approach implemented onsite at a large European industrial manufacturing company in Germany.

### Principal Results

Given the comparable rates of successful communication of test results in both the study groups, our data show that the telemedicine-guided self-collection approach for SARS-CoV-2 diagnostics, including registration, swab self-collection, shipping, and communication of SARS-CoV-2 PCR test result, is technically feasible. The ratings of acceptability, ergonomics, and efficacy for the self-collection approach were noninferior compared with those of the regular testing approach, with the limitation that the ratings were based on different questions for groups 1 and 2 (see Table S2 in Multimedia Appendix 1). Furthermore, the self-collection approach was rated as significantly less time-consuming than the regular testing approach. Neither gender nor age had an effect on the uncertainty regarding the study results or the acceptability, ergonomics, and efficiency ratings. Moreover, a favorable rating for acceptance and efficacy did not affect the outcome and performance of the study procedures, suggesting that a telemedicine-guided self-collection approach for SARS-CoV-2 diagnostics can be applied even among less motivated individuals.

Intriguingly, none of the 293 SARS-CoV-2 PCR tests returned positive. This might be ascribed to a low pretest probability, because all the employees were invited to participate, independent of any prevalent risk contacts or COVID-19 symptoms. Furthermore, the diagnostic laboratory was working at capacity during the second wave of the pandemic, and specimens from patients were prioritized, leading to a delay of study-specific diagnostics and the possible degradation of viral nucleic acid.

Our simulation results suggest that both the testing approaches have the potential to reduce the risk of infection posed by infectious but undetected individuals. Owing to lower sensitivity of self-collected swabs [3], the self-collection approach tends to be slightly less effective than the regular testing approach, whereas the time taken to communicate the test result is the same for both approaches. However, due to the high workload of the virological laboratory at the time of this study, we could not assess the time taken to communicate the test result, hindering a clear assessment of the testing approaches used in this study.

The costs pertaining to the investigated testing approaches vary depending on context-specific factors, including the costs for PCR tests, supply of medical staff, information technology (IT) infrastructure, and onsite time associated with testing. Therefore, a detailed cost evaluation is not within the scope of this study. However, to estimate the costs pertaining to the two testing approaches examined in this study, we conducted back-of-the-envelope calculations for the large central European manufacturing company. Interestingly, the costs of the two testing approaches mainly differ with respect to the five types of costs: costs for medical staff, hygiene costs, productivity loss, shipping costs, and IT costs. The proportion of these five types of costs indicates the testing approach that is more cost effective. The regular testing approach showed higher costs for medical staff, hygiene, and productivity loss than the self-collection approach. In contrast, the self-collection approach entailed higher shipping costs and costs for setting up and operating the app than the regular testing approach. The main share of the app costs was fixed, however, leading to a decrease in the costs per test in proportion to the number of tests conducted in the case of the self-collection approach. Therefore, the self-collection approach might be more favorable for large testing programs. Our back-of-the-envelope calculations indicate that the self-collection approach can be substantially less expensive than the regular approach for large testing programs. However, we recommend that the companies calculate the cost of testing approaches individually, considering context-specific factors.

### Limitations

This study has several limitations. The age and gender did not significantly differ between the two study cohorts, suggesting an appropriate comparability of the results. However, the male participants outnumbered the female participants, which can be ascribed to a predominantly higher male workforce at the Siemens site. The study cohort did not include participants above 64 years of age. The questionnaires on user satisfaction were not completed by 40 and 41 participants in group 1 and group 2, respectively. In future studies, efforts should be made to increase the questionnaire completion rates. The primary end point was defined both as the successful communication of the test result and the lack of reported uncertainty regarding the test result in the questionnaire (see Table S2 in Multimedia Appendix 1). We note that the user satisfaction questionnaires were not completed by all participants. Therefore, it is possible that few participants might have felt insecure regarding their test results and avoided reporting it. To assess the validity of our results, we repeated our analyses by assuming that the nonresponse to the questionnaire is equivalent to the uncertainty regarding the test results. This approach did not significantly change the results of our primary analysis (see Table S5 in Multimedia Appendix 1). COVID-19 symptoms were less often reported in group 2, which might be attributed to nondisclosure of symptoms at the workplace. The risk factors for COVID-19 were more frequently reported in group 2, which is possibly related to assessment by the medical staff. The responses in the questionnaire for symptoms and risk factors were incompletely transmitted by the app for few participants in group 2. Furthermore, the software app should be upgraded to prevent double registrations. Due to the abovementioned high workload in the virological laboratory at the time the study was performed, turnaround times were delayed, and the results could not be provided in 23 cases (7.8%), which might have affected the satisfaction of those participants. Four participants commented that the alignment of the Likert scale was not clear; however, the interpretation of all the participants was correct. The ambiguity was clarified in the app during the course of the study. Nonetheless, we cannot exclude that the visual slider was occasionally moved inversely.

The simulations conducted to assess the effectiveness of the regarded testing approaches rely on several assumptions that are partially based on the expert ratings of the participating investigators. Therefore, bias cannot be excluded. Furthermore, the simulation model assumed few factors, such as a constant incidence rate and immunity rate within the specific period of 4 weeks. The developed simulation model as well as the developed Shiny app exclusively focuses on the risk of infection posed by undiagnosed but infectious individuals. Superspreading events as well as secondary transmissions by index patients were not considered in this model.

### Comparison With Prior Work

Several recent studies evaluated the diagnostic reliability of self-collected versus professional-collected specimen for the detection of SARS-CoV-2 and found comparable sensitivities [3-7]. To the best of our knowledge, telemedicine-guided self-collection approaches in a home-based setting followed by PCR-based SARS-CoV-2 testing have not been investigated to date.

### Conclusions

This study provides evidence that a telemedicine-guided self-collection approach for SARS-CoV-2 diagnostic testing is technically feasible, and this approach is favorably rated in terms of acceptance, ergonomics, and efficiency. Our data indicate that the resources, expense of time and labor, and personal contacts can be considerably reduced through a telemedicine-guided, self-collection approach when compared with a regular PCR-based testing strategy. Nonetheless, the risk reduction in a self-collection approach is expected to be slightly lower because the test sensitivity of the self-collected swabs is inferior to that of professional-collected swabs. Self-test approaches based on lateral-flow antigen tests may be a cost-effective alternative to PCR-based strategies and should be investigated in future studies because the test sensitivity appears to be secondary to the turnaround time regarding the risk reduction. The app-based platform we provide here may serve as the basis for enhanced connectivity in future digital approaches of personalized medicine. Indeed, the easy-to-use design combined with potential coupling with other health care interfaces may provide benefits beyond the COVID-19 pandemic.

## Appendix

Multimedia Appendix 1 Supplementary figures and tables.

Multimedia Appendix 2 Self-sampling instruction video.

## Abbreviations

CTM: Clinical Virus Transport Medium
IT: information technology
NAAT: nucleic acid amplification test
NASA: National Aeronautics and Space Administration
PCR: polymerase chain reaction
QR: quick response
UN 3373: United Nations’ recommendation of dangerous goods
