# Supplementary material for: A Telemedicine-Guided Self-Collection Approach for PCR-Based SARS-CoV-2 Testing: Comparative Study
Source: JMIR Form Res. 2022 Jan 4;6(1):e32564. doi: 10.2196/32564 (PMC8729873; doi:10.2196/32564)
Supplement: Multimedia Appendix 1 [file formative_v6i1e32564_app1.docx]

**Supplementary File 1** **of the Manuscript:**

**Telemedicine-Guided Self-Collection Approach for PCR-Based SARS-CoV-2 Testing: Comparative Study**

**Contents:**

**Material S1 Advertising flyer in English and German**

**Material S2 Self-collection instructions in English and German**

**Table S1 Symptoms and risk factors of the participants**

**Table S2 Questionnaires in English and German**

**Table S3 Transcribed comments of the participants in German and English**

**Table S4 Threshold values for the analysis of ergonometric evaluation, acceptance, and efficacy**

**Table S5 Assessment of the validity of the results based on the assumption: non-response to the questionnaire is considered equivalent to the uncertainty regarding the test results**

**Material S1. Advertising flyer in English and German**

**
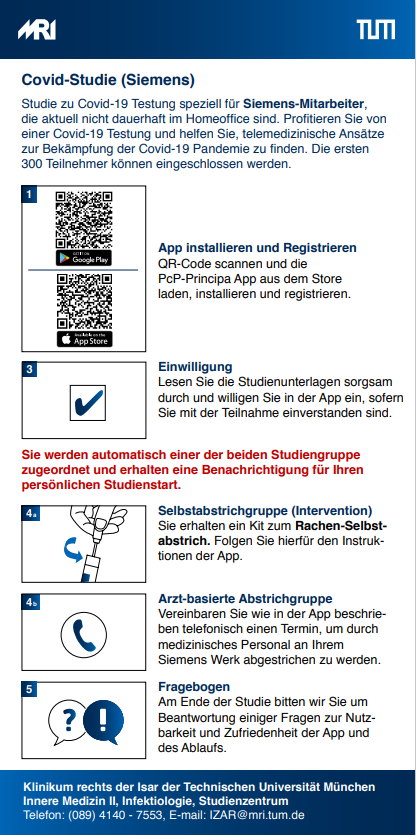
Covid-study (Siemens)**

Study on Covid-19 testing, specifically for Siemens employees who are not permanently working in home office. You may profit from a Covid-19 test and support the development of telemedical approaches to fight the Covid-19 pandemic. The first 300 participants can be enrolled.

**1. App download and registration**

Scan the QR-code and download the Principa App from the store, install, and register.

**3. Consent**

Read the documents carefully and provide your consent within the App if you agree to participate in the study.

You will be automatically assigned to one of the two study groups and receive a notification regarding the commencement of the study.

**4a. Self-collection group (intervention)**

You will be provided with a kit for self-collection of an oropharyngeal swab. Follow the instructions in the App.

**4b. Physician-collected swab group**

Schedule an appointment at the Siemens site through the App to request a health-care professional to collect your swab.

**Questionnaire**

We request you to answer a few questions on the usability and the satisfaction with the App and procedure, at the end of the study.

**Material S2.** **Self-collection instructions in English and German**


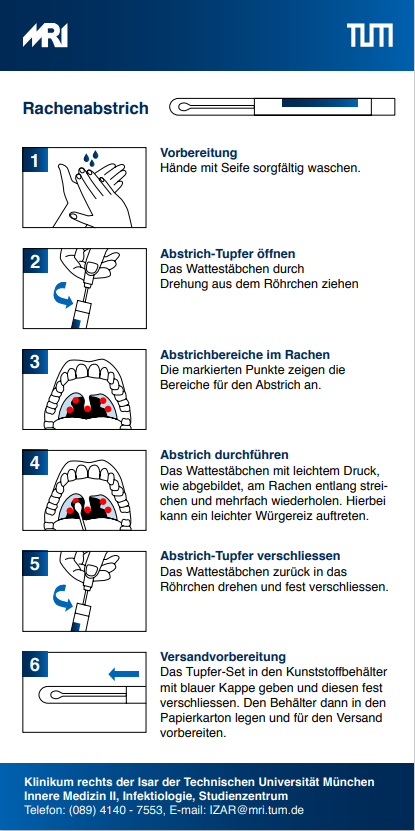


**Oropharyngeal swab**

**1. Preparation**

Wash your hands carefully using soap.

**2. Open swab**

Remove the cotton swab from the tube by rotating the tube.

**3. Area of interest in the throat**

The marked dots indicate the area to be sampled.

**4. Collection of the test**

Rub the cotton swab over the pharyngeal area with light pressure, as depicted, and repeat several times. A gag reflex may occur.

**5. Close the transport tube**

Place the cotton swab back into the tube and ensure that it is tightly closed.

**6. Shipping preparation**

Place the swab set in the plastic container with the blue cap and ensure that it is tightly closed. Thereafter, place the container in the cardboard and prepare it for shipment.

**Table S1. Symptoms and risk factors of the participants**

| **Both groups - symptoms and risk factors:** | | **Group 1; >1 positive answer** | **Group 2; >1 positive answer** | **Both groups; >1 positive answer** |
| --- | --- | --- | --- | --- |
| 1 | Do you currently have any of the following symptoms (multiple answers acceptable)?  a. fatigue  b. tiredness  c. cough  d. shortness of breath  e. rhinitis  f. loss of smell  g. loss of taste  h. sore throat  i. headache  j. limb pain  k. shivering  l. diarrhea  m. elevated temperature  n. fever (≥ 38°C)  *Haben Sie eines oder mehrere der folgenden Symptome aktuell (Mehrfachnennung möglich): a. Abgeschlagenheit b. Müdigkeit c. Husten d. Kurzatmigkeit e. Schnupfen f. Riechminderung g. Geschmacksverlust h. Halsschmerzen i. Kopfschmerzen j. Gliederschmerzen k. Frösteln l. Durchfall m. Erhöhte Temperatur n. Fieber (ab 38°C)?* | 49 (51%); 20 (21%) | 19 (29%); 1 (2%) | 68 (42%); 21 (13%) |
| 2 | Did you experience at least one of the following symptoms within the last 48 hours (multiple answers acceptable):  a. fatigue  b. tiredness  c. cough  d. shortness of breath  e. rhinitis  f. loss of smell  g. loss of taste  h. sore throat  i. headache  j. limb pain  k. shivering  l. diarrhea  m. elevated temperature  n. fever (≥ 38°C)  *Hatten Sie eines oder mehrere der folgenden Symptome in den letzten 48 Stunden (Mehrfachnennung möglich): a. Abgeschlagenheit b. Müdigkeit c. Husten d. Kurzatmigkeit e. Schnupfen f. Riechminderung g. Geschmacksverlust h. Halsschmerzen i. Kopfschmerzen j. Gliederschmerzen k. Frösteln l. Durchfall m. Erhöhte Temperatur n. Fieber (ab 38°C)?* | 57 (59%); 20 (21%) | 16 (25%); 2 (3%) | 73 (45%); 22 (14%) |
| 3 | Did you have any of the following symptoms within the last 3–14 days (multiple answers acceptable):  a. fatigue  b. tiredness  c. cough  d. shortness of breath  e. rhinitis  f. loss of smell  g. loss of taste  h. sore throat  i. headache  j. limb pain  k. shivering  l. diarrhea  m. elevated temperature  n. fever (≥ 38°C)  *Hatten Sie eines oder mehrere der folgenden Symptome in den letzten 3-14 Tagen (Mehrfachnennung möglich): a. Abgeschlagenheit b. Müdigkeit c. Husten d. Kurzatmigkeit e. Schnupfen f. Riechminderung g. Geschmacksverlust h. Halsschmerzen i. Kopfschmerzen j. Gliederschmerzen k. Frösteln l. Durchfall m. Erhöhte Temperatur n. Fieber (ab 38°C)?* | 63 (66%); 31 (32%) | 19 (29%); 2 (3%) | 82 (51%); 33 (20%) |
| 4 | Which of the following risk factors apply to you (multiple answers acceptable)?  a. smoking  b. past smoking  c. cardiovascular diseases  d. diabetes mellitus  e. immunosuppressive therapy  f. immune deficiency  *Bestehen bei Ihnen einer oder mehrere der folgenden Risikofaktoren: (Mehrfachnennung möglich): a. Aktives Rauchen b. Früheres Rauchen c. Herz-, Kreislauferkrankungen d. Diabetes mellitus (Blutzuckererkrankung) e. Immunsuppressive Therapie f. Immunschwäche-Erkrankung?* | 42 (44%); 6 (6%) | 39 (60%); 8 (12%) | 81 (50%); 14 (9%) |

**Table S2. Questionnaires in English and German**

Participants used a visual slider on a 7-point Likert scale to evaluate the different statements upon receiving the test result (0 = strong disagreement, 6 = strong agreement). The median, minimum, and maximum of all the ratings is displayed for both the groups.

To evaluate the primary endpoint of the study, the uncertainty is defined as follows:

Group 1: Statement No. 30 (“I understand the test result and the subsequent consequences.”) was rated below 4 points and/or statement No. 33 (“I feel left alone with my test result not being able to ask individual questions.”) was rated above 2 points on the Likert scale.

Group 2: Statement No. 25 (“I understood the test report communicated over call and its subsequent consequences.”) was rated below 4 points and/or statement No. 27 (“I feel left alone with the test result provided by phone.”) was rated above 2 points on the Likert scale.

| **No.** | **Statement** |  |
| --- | --- | --- |
| **Group 1** | | **Median (Range)** |
| 1 | Registration and installation of the App was convenient. //  *Die Einrichtung der App und die Anmeldung in der App funktionieren sehr leicht.* | 5 (0 – 6) |
| 2 | Using the App for the first time is convenient. //  *Die App das erste Mal zu benutzen ist einfach.* | 5 (1 – 6) |
| 3 | The App is straightforward and well designed. //  *Die App kommt mir übersichtlich und gut strukturiert vor.* | 5 (1 – 6) |
| 4 | Navigating within the App is effortless. //  *In der App zu navigieren ist einfach.* | 6 (1 – 6) |
| 5 | I understood the instructions on data-protection regulation. //  *Ich habe die Hinweise zum Datenschutz gut verstanden.* | 6 (1 – 6) |
| 6 | It is difficult to navigate within the App. //  Es ist schwierig, sich in der App zurecht zu finden. | 1 (0 – 6) |
| 7 | Further explanations are required for using the App. //  *Die App benötigt mehr Erklärungen.* | 1 (0 – 6) |
| 8 | The explanatory video on the throat swab is understandable. //  *Das Video zur Anleitung der Rachenabstriche ist gut verständlich.* | 6 (4 – 6) |
| 9 | The video on the throat swab should be explained in more detail. //  *Das Video zur Anleitung der Rachenabstriche sollte ausführlicher sein.* | 0 (0 – 6) |
| 10 | The video on the packaging of the sampling material is understandable. //  *Das Video zur Verpackung des Probenmaterials ist gut verständlich.* | 6 (0 – 6) |
| 11 | The video on the packaging of the sampling material should be more detailed. //  *Das Video zur Verpackung des Probenmaterials sollte ausführlicher sein.* | 1 (0 – 6) |
| 12 | The self-collected SARS-CoV-2 swabs for at-home use compared to swabs taken by a physician are of great interest to me. //  *Die selbst durchgeführten SARS-CoV-2 Abstriche für zuhause sind gegenüber einem externen Abstrich durch einen Arzt von großem Interesse für mich.* | 5 (1 – 6) |
| 13 | I prefer the self-collection of the swabs for SARS-CoV-2 testing and mailing the swab to scheduling an appointment at a test center and being tested by a health-care professional. //  *Ich würde einen SARS-CoV-2 Selbstabstrich zuhause und den Rückversand per Post gegenüber der Vereinbarung eines Termins bei einem medizinischen Mitarbeiter sowie dem Abstrich durch in einem Testzentrum durch einen Arzt / medizinischen Mitarbeiter bevorzugen.* | 5 (0 – 6) |
| 14 | I feel more flexible with the self-collection of the swab for SARS-oV-2 testing in terms of the time, compared with the collection of the swab by a health-care professional at a test center. //  *Der SARS-CoV-2 Selbstabstrich ermöglicht mir eine höhere zeitliche Flexibilität im Vergleich zum Abstrich durch einen Arzt / medizinischen Mitarbeiter in einem Testzentrum.* | 6 (1 – 6) |
| 15 | The self-collection of swabs for SARS-CoV-2 testing is less time-consuming compared with the collection of the swab by a health-care professional at a test center. //  *Die SARS-CoV-2 Selbstabstriche benötigen weniger zeitlichen Aufwand im Vergleich zum Abstrich durch einen Arzt / medizinischen Mitarbeiter in einem Testzentrum.* | 6 (0 – 6) |
| 16 | The self-collection of swabs for SARS-CoV-2 testing seems to be more secure to me in terms of the infection risk (considering the exposure on the way to a test center and at the test center), compared with the collection of the swab by a health-care professional at a test center. //  *Die SARS-CoV-2 Selbstabstriche erscheinen mir in Bezug auf eine potentielle Ansteckungsgefahr (auf dem Weg ins Testzentrum, im Testzentrum selbst) sicherer im Vergleich zum Abstrich durch einen Arzt / medizinischen Mitarbeiter in einem Testzentrum.* | 5 (0 – 6) |
| 17 | The self-collection of swabs for SARS-CoV-2 testing is less comfortable than the collection of swabs by a health-care professional at a test center. //  *Ich stelle mir einen SARS-CoV-2 Selbstabstrich weniger unangenehm vor im Vergleich zu einem Abstrich durch einen Arzt / medizinischen Mitarbeiter.* | 5 (0 – 6) |
| 18 | Performing the self-collection of swabs for SARS-CoV-2 testing was simple and successful from my point of view. //  *Der SARS-CoV-2 Selbstabstrich war leicht durchzuführen und aus meiner Sicht erfolgreich.* | 5 (1 – 6) |
| 19 | I am happy with the self-collection procedure of the swab for SARS-CoV-2 testing. //  *Ich bin zufrieden mit der Durchführung meines SARS-CoV-2 Selbstabstrichs.* | 6 (0 – 6) |
| 20 | Self-collection of the swab for SARS-CoV-2 testing requires immense mental effort because the task is complex. Self-collection of the SARS-CoV-2 swab is technically challenging. //  *Der SARS-CoV-2 Selbstabstrich erfordert einen hohen geistigen Einsatz, da die Aufgabe komplex ist. Die SARS-CoV-2 Selbstabstriche sind technisch schwierig.* | 1 (0 – 6) |
| 21 | I felt uncertain while performing the self-collection procedure of the SARS-CoV-2 swab. //  *Ich fühlte mich unsicher bei der Durchführung des SARS-CoV-2 Selbstabstrichs.* | 3 (0 – 6) |
| 22 | Performing the self-collection of the swab for SARS-CoV-2 testing frustrated me. //  *Ich bin frustriert von der Durchführung des SARS-CoV-2 Selbstabstrichs.* | 1 (0 – 6) |
| 23 | It is convenient to return the swab tube via mail. //  *Der Rückversand des Abstrichröhrchens per Post ist gut durchführbar.* | 6 (0 – 6) |
| 24 | Overall, the handling of the packaging is appropriate. //  *Die Handhabung der Verpackung ist insgesamt gut.* | 6 (2 – 6) |
| 25 | The packaging of the swab tube is technically difficult. //  *Die Verpackung des Röhrchens ist technisch schwierig.* | 0 (0 – 6) |
| 26 | Packaging of the tube took more than 15 minutes. //  *Ich habe mehr als 15 Minuten gebraucht für die Verpackung des Röhrchens.* | 0 (0 – 6) |
| 27 | The communication of the test result via the App is convenient. //  *Die Kommunikation des Befundes per App ist für mich praktisch.* | 6 (0 – 6) |
| 28 | Finding the test result on the app is effortless. //  *Der Befund ist leicht in der App zu finden.* | 6 (1 – 6) |
| 29 | I prefer the communication of the results via the App to communication via phone. //  *Ich bevorzuge die Ergebniskommunikation per App gegenüber einem Anruf.* | 6 (0 – 6) |
| 30 | I understand the test result and its subsequent consequences. //  *Ich verstehe meinen Befund und die daraus folgenden Konsequenzen.* | 6 (0 – 6) |
| 31 | The written report of the test results available on the App allows me to consider the consequences or the questions regarding the test result. //  *Die schriftliche Kommunikation per App gibt mir Zeit, in Ruhe über die daraus folgenden Konsequenzen oder Fragen nachdenken zu können.* | 5 (1 – 6) |
| 32 | The explanations provided along with the test result on the App are sufficient, and I have no further questions. //  *Die in der App aufgeführten Erklärungen zum Befund reichen mir aus und ich habe keine weiteren Fragen.* | 6 (0 – 6) |
| 33 | I feel left alone with my test result not being able to ask individual questions. //  *Ich fühle mich mit dem Befund, ohne die Möglichkeit individuelle Fragen stellen zu können, allein gelassen.* | 6 (0 – 6) |
| 34 | I have concerns regarding data protection when my test result is communicated via the App. //  *Ich habe Bedenken bei der Mitteilung des Befundes per App bzgl. des Datenschutzes.* | 1 (0 – 6) |
| 35 | Overall, I liked the approach of the self-collection of the swab for SARS-CoV-2 testing and the digital communication of the test result. //  *Das Konzept des SARS-CoV-2 Selbstabstrichs und der digitalen Übermittlung des Ergebnisses per App hat mir insgesamt gut gefallen.* | 6 (1 – 6) |
| 36 | I will be able to manage the self-collection of the swab for SARS-CoV-2 testing in a different scenario similar to this study. //  *Ich könnte mir vorstellen, bei einem erneuten ähnlichen Szenario mit dem Konzept des zugesendeten SARS-CoV-2 Selbstabstrichs wie in der aktuellen Studie gut zurecht zu kommen.* | 6 (1 – 6) |
| 37 | EXPENSE OF TIME in minutes. Please consider the time taken to become acquainted with the App, watch the explanatory video, and pack and ship the sample. //  *ZEITAUFWAND in Minuten. Bitte bedenken Sie hierbei auch an etwaige Zeiten um sich mit der App vertraut zu machen, für das Erklär-Video und das Verpacken und den Versand der Probe.* | Mean 23 (5 – 90) |
| **Group 2** | |  |
| 1 | Registration and installation of the App was convenient. //  *Die Einrichtung der App und die Anmeldung in der App funktionieren sehr leicht.* | 5 (0 – 6) |
| 2 | Using the App for the first time is convenient. //  *Die App das erste Mal zu benutzen ist einfach.* | 5 (0 – 6) |
| 3 | The App is straightforward and well designed. //  *Die App kommt mir übersichtlich und gut strukturiert vor.* | 5 (1 – 6) |
| 4 | Navigating within the app is effortless. //  *In der App zu navigieren ist einfach.* | 6 (1 – 6) |
| 5 | I understood the instructions on data-protection regulation. //  *Ich habe die Hinweise zum Datenschutz gut verstanden.* | 6 (1 – 6) |
| 6 | It is difficult to navigate within the App. //  Es ist schwierig, sich in der App zurecht zu finden. | 1 (0 – 6) |
| 7 | Further explanations are required for using the App. //  *Die App benötigt mehr Erklärungen.* | 2 (0 – 6) |
| 8 | The scheduling of the appointment for swab collection by a health-care professional for SARS-CoV-2 testing was convenient. //  *Ich habe den Termin zum SARS-CoV-2 Abstrich durch einen medizinischen Mitarbeiter ohne Probleme vereinbaren können.* | 6 (0 – 6) |
| 9 | It was time-consuming to schedule the appointment for the collection of the swab for SARS-CoV-2 testing. //  *Es war zeitraubend, den Termin zum SARS-CoV-2 Abstrich vereinbaren zu können.* | 1 (0 – 6) |
| 10 | The self-collected SARS-CoV-2 swabs for at-home use compared to swabs taken by a physician are of great interest to me. //  *Die selbst durchgeführten SARS-CoV-2 Abstriche für zuhause sind gegenüber einem externen Abstrich durch einen Arzt von großem Interesse für mich.* | 4 (0 – 6) |
| 11 | I prefer the self-collection of the swabs for SARS-CoV-2 testing and mailing the swab to scheduling an appointment at a test center and being tested by a health-care professional. //  *Ich würde einen SARS-CoV-2 Selbstabstrich zuhause und den Rückversand per Post gegenüber der Vereinbarung eines Termins bei einem medizinischen Mitarbeiter sowie dem Abstrich durch in einem Testzentrum durch einen Arzt / medizinischen Mitarbeiter bevorzugen.* | 3 (0 – 6) |
| 12 | I feel more flexible with the self-collection of the swab for SARS-oV-2 testing in terms of the time, compared with the collection of the swab by a health-care professional at a test center. //  *Der SARS-CoV-2 Selbstabstrich ermöglicht mir eine höhere zeitliche Flexibilität im Vergleich zum Abstrich durch einen Arzt / medizinischen Mitarbeiter in einem Testzentrum.* | 5 (0 – 6) |
| 13 | The self-collection of swabs for SARS-CoV-2 testing is less time-consuming compared with the collection of the swab by a health-care professional at a test center. //  *Die SARS-CoV-2 Selbstabstriche benötigen weniger zeitlichen Aufwand im Vergleich zum Abstrich durch einen Arzt / medizinischen Mitarbeiter in einem Testzentrum.* | 5 (0 – 6) |
| 14 | The self-collection of swabs for SARS-CoV-2 testing seems to be more secure to me in terms of the infection risk (considering the exposure on the way to a test center and at the test center), compared with the collection of the swab by a health-care professional at a test center. //  *Die SARS-CoV-2 Selbstabstriche erscheinen mir in Bezug auf eine potentielle Ansteckungsgefahr (auf dem Weg ins Testzentrum, im Testzentrum selbst) sicherer im Vergleich zum Abstrich durch einen Arzt / medizinischen Mitarbeiter in einem Testzentrum.* | 3 (0 – 6) |
| 15 | The self-collection of swabs for SARS-CoV-2 testing is less comfortable than the collection of swabs by a health-care professional at a test center. //  *Ich stelle mir einen SARS-CoV-2 Selbstabstrich weniger unangenehm vor im Vergleich zu einem Abstrich durch einen Arzt / medizinischen Mitarbeiter.* | 3 (0 – 6) |
| 16 | The scheduled appointment could be conveniently incorporated in my daily routine. //  *Der angebotene Termin war in meinem zeitlichen Alltag gut integrierbar.* | 6 (1 – 6) |
| 17 | I was able to schedule a timely appointment for the swab collection at a test center for SARS-CoV-2 testing. //  *Ich habe einen zeitnahen Termin zum SARS-CoV-2 Abstrich in einem Testzentrum vereinbaren können.* | 6 (1 – 6) |
| 18 | I had to wait for a long time for my SARS-CoV-2 test appointment. //  *Ich musste lange auf meinen Termin zum SARS-CoV-2 Abstrich warten.* | 1 (0 – 6) |
| 19 | Overall, the appointment for the collection of the SARS-CoV-2 swab consumed significantly more time than expected. //  *Der Termin zur Durchführung des SARS-CoV-2 Abstrichs hat insgesamt deutlich mehr Zeit in Anspruch genommen, als erwartet.* | 0.5 (0 – 6) |
| 20 | The collection of the swab for SARS-CoV-2 testing by a physician/ healthcare professional was seamless. //  *Die Durchführung des SARS-CoV-2 Abstrichs durch einen Arzt / medizinischen Mitarbeiter verlief problemlos.* | 6 (0 – 6) |
| 21 | I am happy with the swab-collection procedure for SARS-CoV-2 testing. //  *Ich bin zufrieden mit der Durchführung meines SARS-CoV-2 Abstrichs.* | 6 (1 – 6) |
| 22 | The collection of the nasopharyngeal swab for SARS-CoV-2 testing was uncomfortable. //  *Die Durchführung des SARS-CoV-2 Nasenrachenabstrichs war unangenehm.* | 4 (0 – 6) |
| 23 | The communication of the test result over call was seamless. //  *Die Befundmitteilung per Telefon hat ohne Probleme funktioniert.* | 6 (1 – 6) |
| 24 | I was not available on phone; therefore, I received the call regarding my test result with a delay. //  *Ich war per Telefon nicht immer erreichbar, daher erreichte mich der Anruf zur Befundübermittlung zeitlich verzögert.* | 1 (0 – 6) |
| 25 | I understood the test report communicated over call and its subsequent consequences. //  *Ich habe den mündlichen Befund sowie die daraus folgenden Konsequenzen verstanden.* | 6 (0 – 6) |
| 26 | I wished to receive a written report for considering the result and its consequences and the questions. //  *Ich würde mir einen schriftlichen Befund wünschen, um über den Befund und daraus folgende Konsequenzen und potentielle Fragen meinerseits nochmals in Ruhe nachdenken zu können.* | 4 (0 – 6) |
| 27 | I feel left alone with the test result provided by phone. //  *Ich fühle mich mit dem telefonischen Befund alleine gelassen.* | 1 (0 – 6) |
| 28 | I would like the approach of the self-collection of the swab for SARS-CoV-2 testing and the digital communication of the test result, as performed in the other study group. //  *Der Ansatz des SARS-CoV-2 Selbstabstrichs und der digitalen Übermittlung des Ergebnisses per App in der anderen Gruppe würde mir insgesamt gut gefallen.* | 5 (0 – 6) |
| 29 | I think I would be able to manage the self-collection of the swab for SARS-CoV-2 testing in a different scenario similar to the approach of the other group in this study. //  *Ich könnte mir vorstellen, bei einem erneuten ähnlichen Szenario mit dem Konzept des zugesendeten SARS-CoV-2 Selbstabstrichs wie in der aktuellen Studie in der anderen Studiengruppe gut zurecht zu kommen.* | 5 (1 – 6) |
| 30 | EXPENSE OF TIME in minutes. Please consider the time taken for scheduling the appointment, commuting to the test center, and for collecting the sample. //  *ZEITAUFWAND in Minuten. Bitte bedenken Sie hierbei auch an etwaige Zeiten für die Terminierung der Probenentnahme, die Anfahrt zum und die Abfahrt vom Testzentrum und die Probenentnahme selbst.* | Mean 38 (3 – 180) |

**Table S3. Transcribed comments of the participants in German and English**

| **Comment (German)** | **Comment (English)** |
| --- | --- |
| Rachenabstrich selber zu machen fand ich sehr challenging. | Self-collection of the throat swab was extremely challenging. |
| Bitte machen Sie weiter so. Nachdem die App auf meinem Huawei P 20 Lite nicht funktioniert hat, gehe ich davon aus das die WEB Version das gleiche Anwender Interface zeigt. Danke das ich teilnehmen durfte. | Keep it up! The app did not work on my Huawei P 20 Lite; I assume that the web version displays the same interface. Thank you for the chance for participation. |
| Paket zu hoch für Postkasten Einwurf. Bitte Höhe reduzieren. Ist zusätzliche Umverpackung des Röhrchens wirklich notwendig? Bitte Kunststoffmüll reduzieren. Proben Zuordnung via App nicht intuitiv. Umweg über Fragebogen notwendig. | The package is too thick to be inserted through a letter box. Please reduce the height. Is the additional outer packaging of the tube indeed necessary? Please reduce plastic waste. Sample assignment via App is not intuitive. Detour via questionnaire necessary. |
| Den größte Zeitaufwand nahm die Organisation und Fahrt zur Post inanspruch. Zeitaufwand zur Abholung des Test keine, da in diesem Falle „auf der Arbeit“. | Organizing and commuting to the post office were the most time consuming processes. Picking up the test kit took no time, as it was available “at work.” |
| Ich fände eine Benachrichtigung der App selbst nach Eingang des Testergebnisse gut. | I would like to be notified through the App upon the arrival of the test result. |
| Die Überwindung des Brechreizes ist für mich die größte Frage, ob dadurch das Ergebnis verfälscht werden kann. | The most important question I have is whether the elimination of the gag reflex can distort the result. |
| Ich fand den Abstrich extrem unangenehm durchzuführen. Die Erläuterungen zum Abstrich und Verpackung waren gut, zum Scannen der Nummer schlecht. Auch die Erläuterungen zu den Symptomen waren schlecht. | Performing the swab was extremely uncomfortable. The explanations for the swab and packaging were adequate; however, the explanation pertaining to scanning the number and symptoms were not appropriate. |
| Die Verpackung sollte schmaler sein, damit sie in die schicken gelben Postkaesten der Post passt. Sonst muss man zur Abgabe in die Filiale. | The packaging should be thinner, such that they fit into the fancy yellow mail boxes of the postal service. Otherwise, one has to mail them via a post office. |
| Sehr gutes Konzept und tolle Anleitung. | Excellent concept and instruction. |
| Bitte die App intuitiver machen. Das ist für mich der entscheidende Punkt bei der Digitalisierung. | Please make the App more intuitive. To me, this is the crucial point in terms of digitalization. |
| Die Übermittlung des Ergebnisses hat sehr lange gedauert. Dadurch keine wirkliche Sicherheit da man sich in der Zwischenzeit leicht angesteckt haben kann. | The transmission of the test result took an extremely long time. Therefore, there is no certainty because one could get infected in the meantime. |
| Es war nicht ganz ersichtlich, ob das Porto bereits bezahlt wurde. | It was not quite clear whether the postal charges had already been paid. |
| Fand das sehr einfach und unkompliziert, würde es jederzeit wieder machen. Ich finde es wichtig zu testen, je mehr getestet wird umso besser, sollte noch mit der corona warm App verknüpft warden. | It is easy and uncomplicated, and I would do it again any time. I think it is important to test; more the number of tests performed, the better it is; it should also be connected with the “Corona Warn App.” |
| Der Selbstabstrich ist doch gewöhnungsbedürftig in der Durchführung. Evtl. ist eine Durchführung von Fachpersonal schneller/besser, bedeutet aber sehr viel mehr organisatorischen/zeitlichen Aufwand. | One needs to become acquainted with the procedure of self-collection of the swab; the collection of the swab by professionals may be faster/better, although it implies more organizational/time expense. |
| Der selbsttest ist sehr unangenehm. Es ist angenehmer wenn eine zweite Person den Test macht da man sich am Anfang sehr überwinden muss das Stäbchen bis in den Rachen einzuführen. | The self-test is extremely uncomfortable. It is more comfortable if a second person performs the test because one has to insert the swab to the pharynx. |
| Top. | Perfect. |
| Die Registrierung der App war etwas problematisch ("Bestätigung in zwei Schritten aktiveren" funktionierte nicht). | Registration via the App was slightly difficult (“Activate confirmation in two steps” was not working). |
| Ist ein Rachenabstrich ausreichend während oft Rachen- und Nasenabstriche gemacht werden? | Is a throat swab sufficient, given that combined throat and anterior nasal swabs are often performed? |
| Die Bewertungsskala war nicht erläutert. Annahme 0 ist keine Zustimmung, 6 ist volle Zustimmung. | The rating scale was not explained. Assumption: 0 implies no agreement, 6 implies full agreement. |
| Danke . | Thank you. |
| Im Bedarfsfall wäre eine Integration der Testergebnisse in die offizielle Corona-App wünschenswert. | If possible, integration of the test results within the official Corona-app would be suitable. |
| Entscheidendes Problem beim selbständigen Abstrich ist die Unsicherheit ob der Abstrich korrekt vorgenommen wurde und auch Aussage kräftig ist. | A critical problem regarding the self-collected swab is the uncertainty regarding the appropriate collection of the swab and the validity of the result. |
| Da mein Test negativ ist, kann ich keine Antwort bezgl. weitergehende Konsequenzen machen. | Because I have tested negative, I cannot give an answer regarding the further consequences. |
| Das meiste ist zu klein geschrieben. Die unter „Einstellungen“ gemachte Einstellung, dass die Schrift größer angezeigt wird, wird nicht berücksichtigt. | Most of the text was extremely small. The option to display the text in a larger font size provided under “settings” is inoperative. |
| Testergebnisse hat sehr lange gedauert, außerdem war dieser Fragebogen erst ein/zwei Wochen nach dem Test verfügbar. | Test results took an extremely long time; further, this questionnaire was only available one/two weeks after the test was conducted. |
| Ich hätte mir eine zeitnahe !Benachrichtigung gewünscht. Eine Woche = 30.11. später eine email zu erhalten, interessiert mich nicht mehr. Jetzt steht das Ergebnis mit Datum 27.11. drin - das stimmt sicher nicht! Bis 29.11. habe ich täglich nachgesehen. | I would have wished for a timely notification. Receiving an email one week (30/11) later is of no interest to me. Now, the result is dated 27/11 – this is definitely not correct! I checked every day until 29/11. |
| 2Fa Fehler bei erstanmeldung. Hinweis in der Anleitung (keine autovervollständigung) unzureichend | 2Fa error upon first-time registration! The advice under instructions (no auto-fill) is insufficient. |
| Super Initiative! Ich bin davon überzeugt, dass ein Sebsttest eine gute Alternative für bestimmte Personengruppen darstellt und zu einer Entlastung des med. Personals führen kann. | Extraordinary initiative! I am convinced that self-testing is an appropriate alternative for certain groups of people and can reduce the workload of the medical personnel. |
| Super Sache. Push-Benachrichtigung kam aber nicht. | Excellent! However, push notification was not received. |
| Hat prima geklappt. Hätte mir gewünscht als Homeofficler nicht extra in den Betrieb fahren zu müssen. Aber war ok. Alles top. Die App sollte noch etwas UI technisch überarbeitet werden. Ansonsten ganz schick. Danke sehr. | It worked out smoothly. Because I am working from home, I would have preferred to not have to drive to the office. Nonetheless, it was all right. Everything is perfect. The UI of the app should be revised. Otherwise, it is great, overall. Thank you. |
| Die Rückmeldung könnte etwas zügiger sein. Wenn man wirklich auf einen Test angewiesen ist, wäre mir die Zeitdauer zu lang und ich würde einen Schnelltest vor Ort durchführen. | The response could be faster. If I would actually need to perform the test, I would perform a rapid test on site because the time consumed is extremely long. |

**Table S4. Threshold values for the analysis of ergonometric evaluation, acceptance, and efficacy**

The marked statements were considered for the evaluation of the respective secondary endpoint. Threshold values were defined to transfer the ratings into a dichotomous scale (favorable rating >3 point; unfavorable rating ≤3 point).

| **Ergonomics** | **Acceptability** | **Efficacy** | **Threshold value for evaluation as agreement”** | **Statement** |
| --- | --- | --- | --- | --- |
| **Group 1** | | | | |
| x |  |  | >3 | Registration and installation of the App was convenient. |
| x |  |  | >3 | Using the App for the first time is convenient. |
| x |  |  | >3 | The App is straightforward and well designed. |
| x |  |  | >3 | Navigating within the App is effortless. |
| x |  |  | >3 | I understood the instructions on data-protection regulation. |
| x |  |  | ≤3 | It is difficult to navigate within the App. |
| x |  |  | ≤3 | Further explanations are required for using the App. |
| x |  |  | >3 | The explanatory video on the throat swab is understandable. |
| x |  |  | ≤3 | The video on the throat swab should be explained in more detail. |
| x |  |  | >3 | The video on the packaging of the sampling material is understandable. |
| x |  |  | ≤3 | The video on the packaging of the sampling material should be more detailed. |
|  | x |  | >3 | The self-collected SARS-CoV-2 swabs for at-home use compared to swabs taken by a physician are of great interest to me. |
|  | x |  | >3 | I would prefer the self-collection of the swabs for SARS-CoV-2 testing and mailing of the swab to scheduling an appointment at a test center and being tested by a health-care professional. |
|  |  | x | >3 | I feel more flexible with the self-collection of the swab for SARS-oV-2 testing in terms of the time, compared with the collection of the swab by a health-care professional at a test center. |
|  |  | x | >3 | The self-collection of swabs for SARS-CoV-2 testing is less time-consuming compared with the collection of the swab by a health-care professional at a test center. |
|  | x |  | >3 | The self-collection of swabs for SARS-CoV-2 testing seems to be more secure to me in terms of the infection risk (considering the exposure on the way to a test center and at the test center), compared with the collection of the swab by a health-care professional at a test center. |
|  | x |  | >3 | The self-collection of swabs for SARS-CoV-2 testing is less comfortable than the collection of swabs by a health-care professional at a test center. |
| x |  | x | >3 | Performing the self-collection of swabs for SARS-CoV-2 testing was simple and successful from my point of view. |
|  | x |  | >3 | I am happy with the self-collection procedure of the swab for SARS-CoV-2 testing. |
| x |  |  | ≤3 | Self-collection of the swab for SARS-CoV-2 testing requires immense mental effort because the task is complex. Self-collection of the SARS-CoV-2 swab is technically challenging. |
| x |  |  | ≤3 | I felt uncertain while performing the self-collection procedure of the SARS-CoV-2 swab. |
|  | x |  | ≤3 | Performing the self-collection of the swab for SARS-CoV-2 testing frustrated me. |
| x |  |  | >3 | It is convenient to return the swab tube via mail. |
| x |  |  | >3 | Overall, the handling of the packaging is appropriate. |
| x |  |  | ≤3 | The packaging of the swab tube is technically difficult. |
| x |  | x | ≤3 | Packaging of the tube took more than 15 minutes. |
| x | x | x | >3 | The communication of the test result via the App is convenient. |
| x |  |  | >3 | Finding the test result on the app is effortless. |
|  | x |  | >3 | I prefer the communication of the results via the App over communication via phone. |
| x |  |  | >3 | The written report of the test results available on the App allows me to consider the consequences or the questions regarding the test result. |
|  | x |  | >3 | The explanations provided along with the test result on the App are sufficient, and I have no further questions. |
|  | x |  | ≤3 | I have concerns regarding data protection when my test result is communicated via the App. |
|  | x |  | >3 | Overall, I liked the approach of the self-collection of the swab for SARS-CoV-2 testing and the digital communication of the test result. |
|  | x |  | >3 | I will be able to manage the self-collection of the swab for SARS-CoV-2 testing in a different scenario similar to this study. |
|  |  | x | < 38 (mean of time expense Group 2) | EXPENSE OF TIME in minutes. Please consider the time taken to get acquainted with the App, watch the explanatory video, and to pack and ship the sample. |
| **Group 2** | | | | |
| x |  |  | >3 | Registration and installation of the App was convenient. |
| x |  |  | >3 | Using the App for the first time is convenient. |
| x |  |  | >3 | I think the App is straightforward and well designed. |
| x |  |  | >3 | Navigating within the app is effortless. |
| x |  |  | >3 | I understood the instructions on data-protection regulation. |
| x |  |  | ≤3 | It is difficult to navigate within the App. |
| x |  |  | ≤3 | Further explanations are required for using the App. |
| x |  |  | ≤3 | The scheduling of the appointment for swab collection by a health-care professional for SARS-CoV-2 testing was convenient. |
| x |  | x | ≤3 | It was time-consuming to schedule the appointment for the collection of the swab for SARS-CoV-2 testing. |
|  | x |  | >3 | The self-collected SARS-CoV-2 swabs for at-home use compared to swabs taken by a physician are of great interest to me. |
|  | x |  | >3 | I prefer the self-collection of the swabs for SARS-CoV-2 testing and mailing the swab to scheduling an appointment at a test center and being tested by a health-care professional. |
|  |  | x | >3 | I feel the self-collection of the swab for SARS-CoV-2 testing is more flexible in terms of the time, compared with the collection of the swab by a health-care professional at a test center. |
|  |  | x | >3 | The self-collection of swabs for SARS-CoV-2 testing is less time-consuming compared with the collection of the swab by a health-care professional at a test center. |
|  | x |  | >3 | The self-collection of swabs for SARS-CoV-2 testing seems to be more secure to me in terms of the infection risk (considering the exposure on the way to a test center and at the test center), compared with the collection of the swab by a health-care professional at a test center. |
|  | x |  | >3 | The self-collection of swabs for SARS-CoV-2 testing is less comfortable than the collection of swabs by a health-care professional at a test center. |
|  |  | x | >3 | The scheduled appointment could be conveniently incorporated in my daily routine. |
|  |  | x | >3 | I was able to schedule a timely appointment for the swab collection at a test center for SARS-CoV-2 testing. |
|  |  | x | ≤3 | I had to wait for a long time for my SARS-CoV-2 test appointment. |
|  | x | x | ≤3 | Overall, the appointment for the collection of the SARS-CoV-2 swab consumed significantly more time than expected. |
| x |  |  | >3 | The collection of the swab for SARS-CoV-2 testing by a physician/ health-care professional was seamless. |
|  | x |  | >3 | I am happy with the swab-collection procedure for SARS-CoV-2 testing. |
| x |  |  | ≤3 | The collection of the nasopharyngeal swab for SARS-CoV-2 testing was uncomfortable. |
| x |  |  | >3 | The communication of the test result over call was seamless. |
|  |  | x | ≤3 | I was not available on phone; therefore, I received the call regarding my test result with a delay. |
|  | x |  | ≤3 | I wished to receive a written report for considering the result and its consequences and the questions. |
|  | x |  | >3 | I would like the approach of the self-collection of the swab for SARS-CoV-2 testing and the digital communication of the test result, as performed in the other study group. |
|  | x |  | >3 | I think I would be able to manage the self-collection of the swab for SARS-CoV-2 testing in a different scenario similar to the approach of the other group in this study. |
|  |  | x | < 23 (mean of time expense Group 1) | EXPENSE OF TIME in minutes. Please also consider the time for scheduling the appointment, commuting to the test center, and for collecting the sample. |

**Table S5. Assessment of the validity of the results based on the assumption: non-response to the questionnaire was considered equivalent to uncertainty regarding the test results**

The results from the primary analysis are depicted for comparison.

|  | Group 1 | Group 2 | *P* value |
| --- | --- | --- | --- |
| Primary analysis: non-response to the questionnaire is considered to be equivalent to certainty regarding the test result | | | |
| positive primary endpoint | 76.0% (114/150) | 76.9% (110/143) | 0.9614 |
| positive secondary endpoint | 16.7% (25/150) | 14.7% (21/143) | 0.7601 |
| females with positive primary endpoint | 20.2% (23/114) | 18.1% (20/110) | 0.8344 |
| median age with positive primary endpoint (median, range) | 41 (20-61) | 43 (23-63) | 0.3032 |
| Validation analysis: non-response to the questionnaire was considered equivalent to uncertainty regarding the test result | | | |
| positive primary endpoint | 56.7% (85/150) | 55.2% (79/143) | 0.8987 |
| positive secondary endpoint | 36.0% (54/150) | 36.4% (52/143) | 1 |
| females with positive primary endpoint | 17.6% (15/85) | 17.7% (14/79) | 1 |
| median age with positive primary endpoint (median, range) | 39 (20-61) | 43 (23-61) | 0.3192 |
